# Critical behavior of the van der Waals bonded high *T*_*C*_ ferromagnet Fe_3_GeTe_2_

**DOI:** 10.1038/s41598-017-06671-5

**Published:** 2017-07-21

**Authors:** Bingjie Liu, Youming Zou, Shiming Zhou, Lei Zhang, Zhe Wang, Hexuan Li, Zhe Qu, Yuheng Zhang

**Affiliations:** 1 Anhui Key Laboratory of Condensed Matter Physics at Extreme Conditions, High Magnetic Field Laboratory, Chinese Academy of Sciences, Hefei, Anhui 230031 China; 2 University of Science and Technology of China, Hefei, Anhui 230026 China; 3 Hefei National Laboratory for Physical Sciences at Microscale, University of Science and Technology of China, Hefei, Anhui 230026 China

## Abstract

Fe_3_GeTe_2_ is a promising candidate for van der Waals bonded ferromagnet because of its high Curie temperature and the prediction that its ferromagnetism can maintain upon exfoliating down to single layer. Here, we have reported the critical behavior to understand its ferromagnetic exchange. Based on various techniques including modified Arrott plot, Kouvel-Fisher plot, and critical isotherm analysis, a set of reliable critical exponents (*β* = 0.327 ± 0.003, *γ* = 1.079 ± 0.005, and *δ* = 4.261 ± 0.009) has been obtained. The critical behavior suggests a three-dimensional long-range magnetic coupling with the exchange distance decaying as *J*(*r*) ≈ *r* ^−4.6^ in Fe_3_GeTe_2_. The possible origin of three-dimensional magnetic characteristics in van der Waals bonded magnets is discussed.

## Introduction

Since the discovery of the graphene, two-dimensional (2D) materials have generated significant interests in recent year^1–3^. Their amazing physics has inspired extensive research on van der Waals (VDW) bonded heterostructures and application-oriented configurations. VDW bonded magnetic materials are of great interest as building blocks for heterostructures in spin-based information technologies^4, 5^. For example, it has been indicated that the application of VDW magnetic materials in data storage technology could result in several-order of magnitude increase in the recording densities^6^.

For the practical application, the ideal VDW bonded magnetic material should maintain its ferromagnetism upon exfoliating down to single layer and must have a high Curie temperature (*T*_*C*_). Within this context, Fe_3_GeTe_2_, a VDW metallic ferromagnet, has recently attracted significant attention due to its high Curie temperature and the prediction of the important coexistence of ferromagnetic (FM) and metallic properties upon exfoliating down to nanosheets^7, 8^.

Fe_3_GeTe_2_ is a layered material which belongs to the P63/mmc space group^7^. It contains Fe_3_Ge slabs separated by VDW bonded Te layers. The Fe atoms occupy two inequivalent Wyckoff positions, one situated in a hexagonal net in a layer with only Fe atoms and the other covalently bounded in an adjacent layer^9^. Fe_3_GeTe_2_ undergoes a paramagnetic (PM)-FM transition with the Curie temperature as high as 220 K^7^. Electronic correlations and quantum fluctuations have been found to be crucial in determining the magnetism in this compound^10^. In order to understand the nature of the magnetic phase transition in detail, we have investigated its critical behavior, expecting the universality class to which the material belongs to give important clues. It is found that the obtained set of exponents does not belong to any single universality class but lies between 3D Heisenberg model and mean field model. The magnetic exchange distance is found to decay as *J*(*r*) ≈ *r* ^−*4*.*6*^, which is close to that of mean-field model (*r* ^−*4*.*5*^) with long-range interaction.

## Results and Discussion

**Figure 1:**
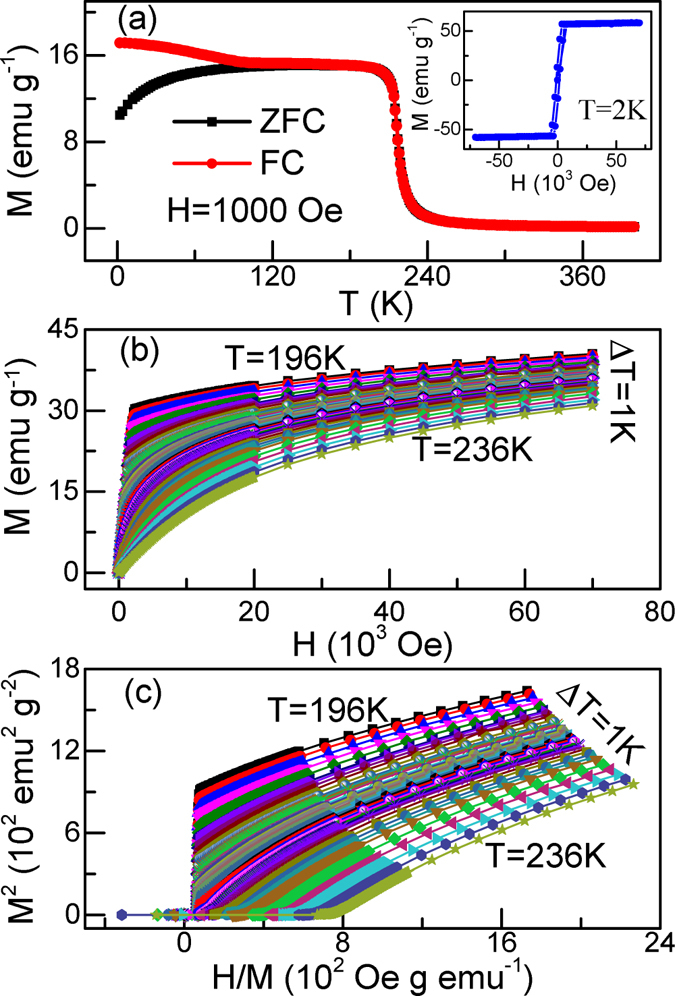
(**a**) Temperature dependence of magnetization *M*(*T*) for Fe_3_GeTe_2_ under *H* = 1000 Oe, the inset shows the isothermal magnetization *M*(*H*) at *2* K. (**b**) Initial magnetization M-H and (**c**) Arrott plots *M* ^*2*^ vs *H*/*M* around *T*_*C*_ for Fe_3_GeTe_2_.

Figure 1(a) shows the temperature dependence of magnetization *M*(*T*) for Fe_3_GeTe_2_ under zero-field-cooling and field-cooling with an applied field of 1000 Oe. An abrupt PM-FM transition can be observed to occur near 220 K. The inset of Fig. 1(a) gives the isothermal magnetization *M*(*H*) at 2 K, which exhibits a typical FM ordering behavior. These results are in good agreement with previous reports^11^. Figure 1(b) and (c) show the isothermal magnetization data around *T*_*C*_ and its Arrott plot, respectively^12^. A positive slope is clearly observed in the Arrott plot, indicating the second-order nature of the PM-FM transition^13^. However, all lines are not parallel to each other, suggesting that Landau mean-field model is not valid for Fe_3_GeTe_2_ and a modified Arrott plot should be used.

**Figure 2:**
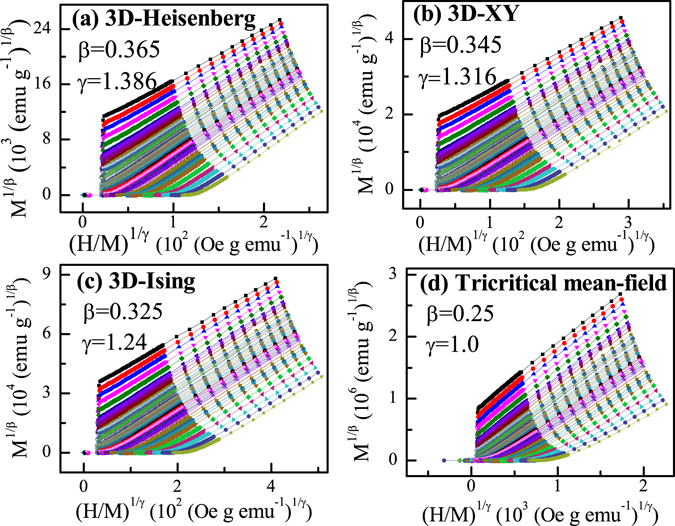
The isotherms of *M* ^*1/β*^ vs.(*H/M*)^*1/γ*^ with (**a**) 3D-Heisenberg model, (**b**) 3D-XY model, (**c**) 3D-Ising model and (**d**) tricritical mean-field model.

The modified Arrott plot (MAP) was then employed to figure out the proper values of critical exponents. For a set of appropriate exponents, the modified Arrott plot should be a series of parallel lines in the high field region with the same slope of *S*(*T*) = *dM* ^*1/β*^ */d*(*H/M*)^*1/γ*^. To obtain an appropriate starting point, we first use four three-dimensional (3D) models, the 3D-Heisenberg model (*β* = 0.365, *γ* = 1.336), 3D-XY model (*β* = 0.345, *γ* = 1.316), 3D-Ising model (*β* = 0.325, *γ* = 1.24), and tricritical mean-field model (*β* = 0.25, *γ* = 1.0) to make MAP^14, 15^. As shown in Fig. 2(a–d), quasi-straight lines are observed in the high field region for all these plots. It can be seen that the lines in Fig. 2(d) are not parallel to each other, suggesting that the tricritical mean-field model is not appropriate to describe the critical behavior of Fe_3_GeTe_2_. However, all lines in Fig. 2(a–c) are almost parallel to each other.

According to the scaling hypothesis^16^, the spontaneous magnetization *M*_*S*_(*T*) below *T*_*C*_, the inverse initial susceptibility *χ*_*0*_^−*1*^(*T*) above *T*_*C*_ and magnetization *M* at *T*_*C*_ can be described with the following mathematical definitions:

1 $${M}_{S}(T)={M}_{0}{(-\varepsilon )}^{\beta },\varepsilon  < 0,T < {T}_{C}$$

2 $${{\chi }_{0}}^{-1}(T)=({h}_{0}/{m}_{0}){\varepsilon }^{\gamma },\varepsilon  > 0,T > {T}_{C}$$

3 $$M=D{H}^{1/\sigma },\varepsilon =0,T={T}_{C}$$

where *ε* = (*T* − *T*_*C*_)*/T*_*C*_ is the reduced temperature; *M*_*0*_, *h*_*0*_ */m*_*0*_ and *D* are the critical amplitudes, respectively.

**Figure 3:**
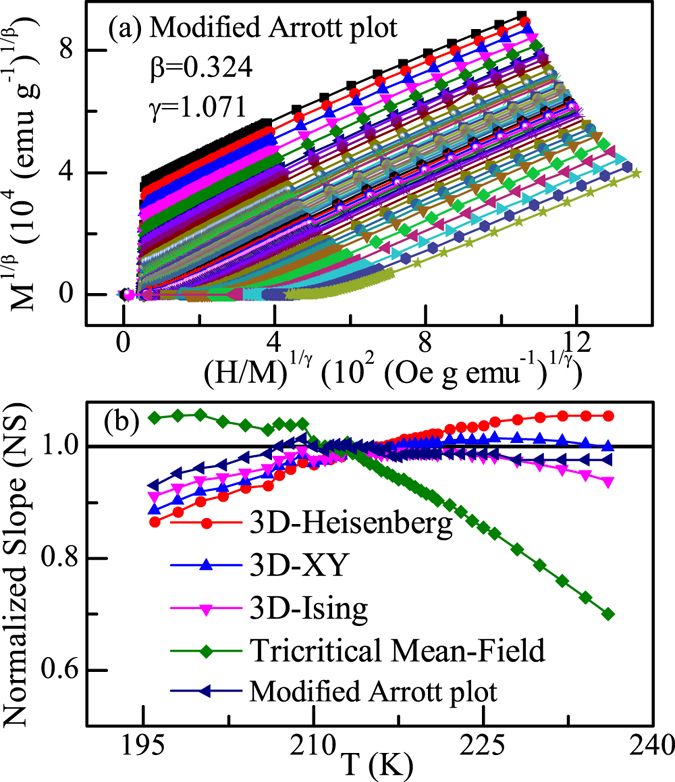
(**a**) Modified Arrott plot of isotherms with *β* = *0*.*324* and *γ* = *1*.*071* for Fe_3_GeTe_2_. (**b**) Normalized slopes (*NS* = *S*(*T*)/*S*(*T*_*C*_)) as a function of temperature with five sets of critical exponents for Fe_3_GeTe_2_.

In order to obtain the proper values of *β* and *γ* for Fe_3_GeTe_2_, a rigorous iterative method was further adopted^17^. The starting values of *M*_*S*_(*T*) and *χ*_*0*_^−*1*^(*T*) are determined from the high field data in 3D-Ising model following Eqs (1) and (2). The obtained new values of *β* and *γ* are then used to figure out new MAP. It should be mentioned that while fitting the straight lines, the free parameter critical temperature *T*_*C*_ is varied to get the best fitting results. This process is repeated until the iterations converge. After doing this exercise, the stable values of exponents, *β* = 0.324 ± 0.002 and *γ* = 1.071 ± 0.005, have been obtained (shown in Fig. 3(a)). It is noted that at low field region, the replotted isotherms are slightly curved as they represent averaging over domains magnetized in different directions^16^. Nevertheless, in high field region, there is a set of good reasonably good parallel straight lines. Moreover, the isotherm is found to pass through the origin at 215.0 K, which is the critical temperature *T*_*C*_ of Fe_3_GeTe_2_. To check which model is the most suitable one, we have calculated the normalized slope *NS* = *S*(*T*)*/S*(*T*_*C*_) and compared them with the ideal value *NS* = 1^18^, which is shown in Fig. 3(b). For the description of the critical behavior of Fe_3_GeTe_2_, the MAP generated by the set of exponents obtained in the iterative method is supreme over other theoretical models. For *T* > *T*_*C*_ *NS* of 3D-XY model is close to unity, while for *T* < *T*_*C*_ the 3D-Ising model is the best. This indicates that the critical behavior of Fe_3_GeTe_2_ may not belong to a single universality class.

**Figure 4:**
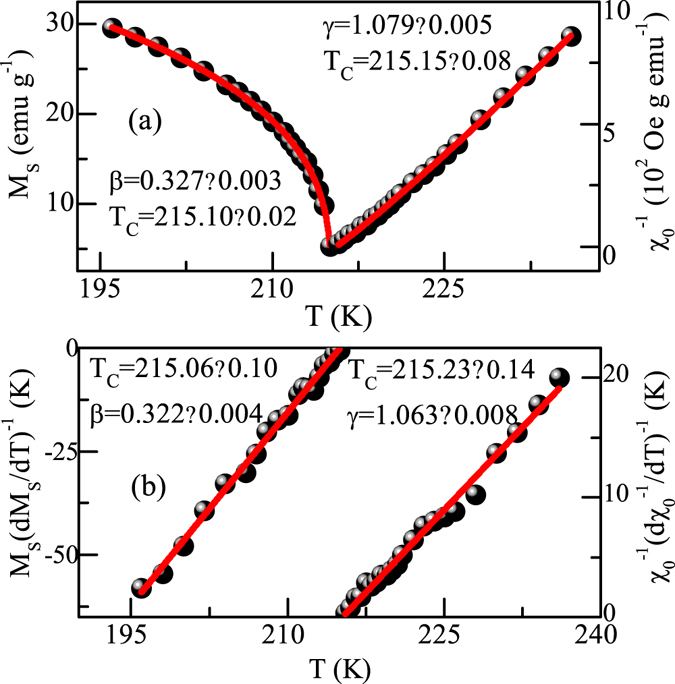
(**a**) Temperature dependence of *M*_*S*_ and *χ*_*0*_^−*1*^ for Fe_3_GeTe_2_ with the fitting solid curves. (**b**) Kouvel-Fisher plot of *M*_*S*_(*dM*_*S*_ */dT*)^−*1*^ and *χ*_*0*_^−*1*^(*dχ*_*0*_^−*1*^ */dT*)^−*1*^ for Fe_3_GeTe_2_ with the fitting solid curves.

The finally obtained *M*_*S*_(*T*) and *χ*_*0*_^−*1*^(*T*) are plotted as a function of temperature in Fig. 4(a). Using the values of *M*_*S*_(*T*) and *χ*_*0*_^−*1*^(*T*), Eq. (1) gives *β* = 0.327 ± 0.003, *T*_*C*_ = 215.10 ± 0.02 K and Eq. (2) gives *γ* = 1.079 ± 0.005, *T*_*C*_ = 215.15 ± 0.08 K, respectively. This estimated critical exponents and *T*_*C*_ are reasonably close to the values obtained from the MAP in Fig. 3(a). We use the Kouvel-Fisher (KF) technique to get a further check of the critical exponents and *T*_*C*_^19^. According to KF method, *M*_*S*_(*dM*_*S*_ */dT*)^−*1*^ and *χ*_*0*_^−*1*^(*dχ*_*0*_^−*1*^ */dT*)^−*1*^ plotted against temperature should be straight lines with slope 1/*β* and 1/*γ*, respectively. As shown in Fig. 4(b), the linear fits to the data yield *β* = *0*.*322* ± *0*.*004*, *T*_*C*_ = 215.06 ± 0.10 K and *γ* = 1.063 ± 0.008, *T*_*C*_ = 215.23 ± 0.14 K. The values of critical exponents and *T*_*C*_ calculated using both MAP and KF plot match reasonably well (see Table 1), suggesting that the obtained values are unambiguous. The difference between these values give an estimate of the uncertainties on these values.

**Table 1:** Comparison of critical exponents of Fe_3_GeTe_2_, CrSiTe_3_ and CrGeTe_3_ with different theoretical models.

Composition	Ref	Technique	β	γ	δ
Fe_3_GeTe_2_	This work	MAP	0.327 ± 0.003	1.079 ± 0.005	4.300 ± 0.045^cal^
		KFP	0.322 ± 0.004	1.063 ± 0.008	4.301 ± 0.065^cal^
		Cricital isotherm			4.261 ± 0.009
Mean field	Ref. 13	Theory	0.5	1.0	3.0
3D Heisenberg	Ref. 15	Theory	0.365	1.386	4.8
3D XY	Ref. 15	Theory	0.345	1.316	4.81
3D Ising	Ref. 15	Theory	0.325	1.24	4.82
Tricritical mean-field	Ref. 18	Theory	0.25	1.0	5
CrSiTe_3_	Ref. 20	MAP	0.170 ± 0.008	1.532 ± 0.001	9.917 ± 0.008
CrGeTe_3_	Ref. 21	MAP	0.242 ± 0.006	0.985 ± 0.003	5.032 ± 0.005

(MAP = modified Arrott plot; KFP = Kouvel-Fisher plot; cal = calculated).

**Figure 5:**
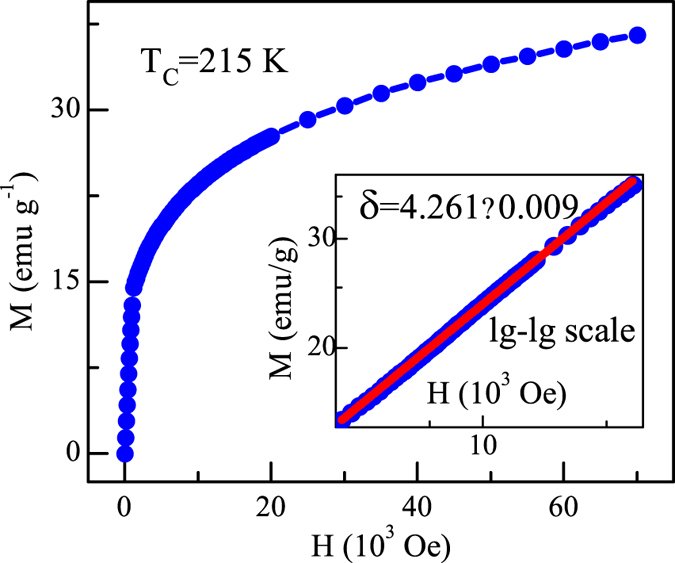
Isothermal *M*(*H*) at *T*_*C*_ with the inset plane on log-log scale for Fe_3_GeTe_2_ (the solid line is fitted).

Figure 5 shows the isothermal magnetization *M*(*H*) at *T*_*C*_ = 215.0 K, with the inset plotted on a log-log scale. According to Eq. (3), the *M*(*H*) at the critical temperature should be a straight line on the log-log scale with the slope *1*/*δ*. Such a fitting yield *δ* = 4.261 ± 0.009. Using the Widom scaling relation *δ* = *1* + *γ*/*β* with the values of *β* and *γ* determined from the MAP and KF plot^16^, we obtain *δ* = 4.300 ± 0.045 and *δ* = 4.301 ± 0.065, respectively, which agree well with the critical isothermal analysis. These results prove that the obtained critical exponents are reliable and accurate within experimental precision.

**Figure 6:**
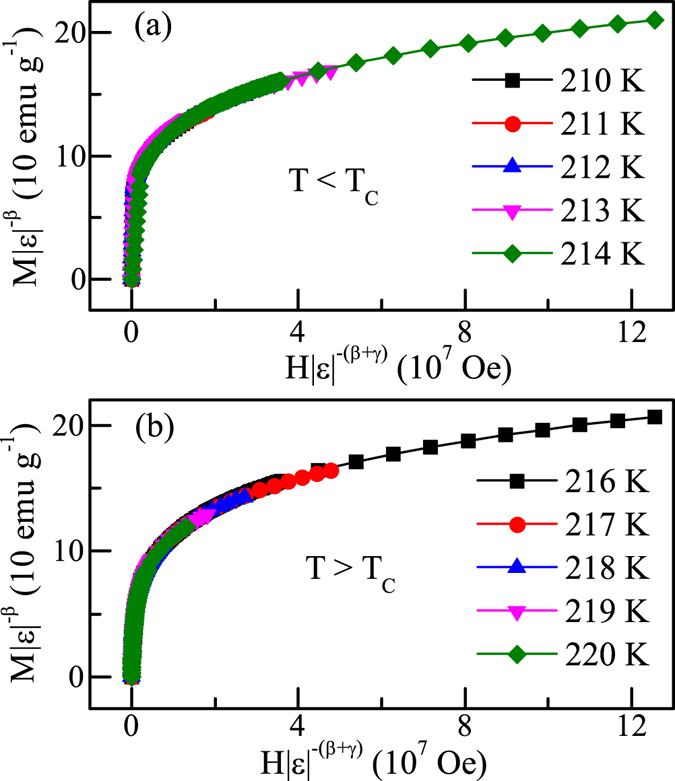
Scaling plots of renormalized magnetization *m* vs renormalized field *h* (**a**) below and (**b**) above the critical temperature for Fe_3_GeTe_2_.

It is important to check whether the obtained critical exponents and *T*_*C*_ can generate a scaling equation of state for this system. According to the scaling hypothesis, in the asymptotic critical region, the magnetic equation is written as^22^:

4 $$M(H,\varepsilon )={\varepsilon }^{\beta }{f}_{\pm }(H/{\varepsilon }^{\beta +\gamma })$$

where *f*_+_ for *T* > *T*_*C*_ and *f*_−_ for *T* < *T*_*C*_, respectively, are the regular functions. Therefore, the renormalized magnetization *m* = *ε* ^−*β*^ *M*(*H*, *ε*) versus *h* = *ε* ^−(*β*+*γ*)^ *H* should follow two universal rules: one for *T* < *T*_*C*_ and the other for *T* > *T*_*C*_. As shown in Fig. 6(a,b), all data collapse into two different curves: one below *T*_*C*_ and another above *T*_*C*_, indicating that the interactions get properly renormalized in critical regime following scaling equation of state.

The critical exponents of Fe_3_GeTe_2_ obtained in this study, along with those of theoretical models are summarized in Table 1. It is seen that the obtained exponents cannot be categorized into any conventional universality classes. The exponent *β* is close to that of 3D-Ising model, which might be the origin of large magnetocrystalline anisotropy in Fe_3_GeTe_2_. While *γ* approaches to that of mean field or tricritical mean field model. It is then important to understand the nature as well as the range of interaction in this material. For a homogeneous magnet, the universality class of the magnetic phase transition depends on the exchange interaction *J*(*r*). A renormalization group theory analysis predicts *J*(*r*) decays with distance *r* as^23^:

5 $$J(r)\approx {r}^{-(3+\sigma )}$$

where *σ* is a positive constant. Moreover, the susceptibility exponent *γ* is predicted as:

6 $$\gamma =1+\frac{4}{d}\frac{n+2}{n+8}{\rm{\Delta }}\sigma +\frac{8(n+2)(n-4)}{{d}^{2}{(n+8)}^{2}}\times [1+\frac{2G(\frac{d}{2})(7n+20)}{(n-4)(n+8)}]{\rm{\Delta }}{\sigma }^{2}$$

where ${\rm{\Delta }}\sigma =(\sigma -\frac{d}{2})$ and $G(\frac{d}{2})=3-\frac{1}{4}{(\frac{d}{2})}^{2}$, *n* is the spin dimensionality. In the present case, it is found that the magnetic exchange distance decays as *J*(*r*) ≈ *r* ^−*4*.*6*^, which should lie between that of the 3D Heisenberg model and the mean-field mode^17, 24, 25^. It is known that short range magnetic exchange interaction contributes to the 3D Heisenberg model, while the mean field model works with a long range magnetic exchange interaction^12^. The magnetic exchange distance is found to decay as *J*(*r*) ≈ *r* ^−*4*.*6*^, which is close to mean-field model (*r* ^−*4*.*5*^) with long-range interaction.

The critical exponents of Fe_3_GeTe_2_ may be compared with those expected for different Hamiltonians and universality classes. Taroni *et al*. have accomplished a comprehensive study of critical exponents values for 2D magnets. They found that the critical exponent *β* for a 2D magnet should lie in *0*.*1* ≤ *β* ≤ *0*.*25* ^26^, which means Fe_3_GeTe_2_ showing 3D critical phenomenon clearly.

At the first sight, it is remarkable and intriguing that a 3D magnetic behavior is observed in a VDW bonded magnet. The 3D magnetic characteristics suggest that the interlayer coupling should not be as weak as the VDW bonding interaction between two adjacent Te layers only. One possibility is that some Fe atoms might occupy the position in the VDW gap, like the case in the isostructural compound Ni_3_GeTe_2_^7, 27^. However, experiment results of X-ray diffraction, Mossbauer spectroscopy and scanning transmission electron microscopy clearly indicate that such an intercalation of Fe is absent in Fe_3_GeTe_2_^27, 28^, suggesting that an alternative mechanism may take effect.

CrXTe_3_ (X = Si, Ge and Sn) and MPS_3_ (M = Mn, Fe, and Ni) are recognized as two major VDW bonded magnetic materials. Chromium Tellurides CrXTe_3_ (X = Si, Ge and Sn) belong to a rare category of ferromagnetic semiconductors possessing a 2D layered structure^29^. Detailed critical analysis and neutron scattering experiments prove that the critical behavior for CrSiTe_3_ falls into the universality class of 2D Ising model^30, 31^. Compared with CrSiTe_3_, CrGeTe_3_ exhibits a smaller VDW gap and a larger cleavage energy, which lead to a transition of critical behavior from 2D Ising to 3D tricritical mean-field model^20^. It is noted that the mean distances (*d*) between two adjacent Te layers that across the VDW gap is 0.374 nm in Fe_3_GeTe_2_^7^, which is much smaller than that of CrSiTe3 *d*_*CrSiTe3*_ = *0*.*423* *nm* and very close to that of CrGeTe_3_ *d*_*CrGeTe3*_ = *0*.*377* *nm* ^21, 32^. The 3D magnetic characteristics might be associated with the smaller VDW gap and higher cleavage energy in Fe_3_GeTe_2_ system.

Transition metal phosphorus trisuflide (or thiophosphate), MPS_3_ (M = Mn, Fe, and Ni), are VDW antiferromagnets. All three principal spin Hamiltonians are reported in these compounds, *i*.*e*. 2D Heisenberg critical behavior in MnPS_3_, 2D XY magnetic behavior in NiPS_3_ and 2D Ising magnetism in FePS_3_^33^. Further neutron measurements indicate that NiPS_3_ undergoes a critical phase transition between 3D and 2D at *T* ∼ *0*.*9T*_*N*_^34^. A similar crossover is also found in MnPS_3_, which is confirmed to 2D anisotropic Heisenberg model for whole range except 3D magnetism just below *T*_*N*_^35^. For Fe_3_GeTe_2_, our critical analysis is restricted in a narrow region around *T*_*C*_ (*|*(*T* − *T*_*C*_)*/T*_*C*_ *|* ≤ *0*.*1*), which suggests the 3D critical behavior observed in Fe_3_GeTe_2_ might be operating similar to that in MPX_3_. Quite recently, it has been reported that the ferromagnetic layers of Fe_3_GeTe_2_ actually order antiferromagnetically along the *c*-axis below 152 K^9^, suggesting a 2D antiferromagnetic (AFM) ground state. Considering the similar 2D AFM ground state at low temperature and 3D critical behavior near phase transition temperature in Fe_3_GeTe_2_ and MPX_3_, it is thus of great interest to investigate whether a critical phase transition from 3D to 2D will occur with decreasing temperature in Fe_3_GeTe_2_ like that in MPX_3_.

## Conclusion

In summary, we have reported a comprehensive study on the critical behavior of the PM-FM phase transition in the high *T*_*C*_ VDW bonded ferromagnet Fe_3_GeTe_2_. We obtain a set of reliable critical exponents by using various techniques including modified Arrott plot, Kouvel-Fisher method, and critical isotherm analysis. The critical exponents obtained from different methods are consistent with each other and show well-obeyed scaling behavior. The set of obtained critical exponents does not belong to any single universality class but lies between 3D Heisenberg model and mean field model. The magnetic exchange distance is found to decay as *J*(*r*) ≈ *r* ^−*4*.*6*^, which is close to that of mean-field model (*r* ^−*4*.*5*^) with long-range interaction. The 3D critical characteristics of Fe_3_GeTe_2_ might be associated with its smaller VDW gap and higher cleavage energy. Further studies are needed to investigate whether a critical phase transition from 3D to 2D will occur with decreasing temperature in Fe_3_GeTe_2_.

## Methods

Single-crystalline sample of Fe_3_GeTe_2_ was prepared by the chemical vapor transport technique^11^. The structure and phase purity were confirmed by single-crystal and powder X-ray diffraction measurements at room temperature. The magnetization was measured using a Quantum Design SQUID-VSM magnetometer with the magnetic field applied parallel to *c* axis of the sample. Isotherms were collected at an interval of 0.5 K around *T*_*C*_. Each curve should be initially magnetized. The applied magnetic field *H*_*a*_ has been corrected by the considering of the demagnetization factor^36^, and the calculated *H* was used for the analysis of critical behavior.
